# Clinical Outcomes between Stand-Alone Zero-Profile Spacers and Cervical Plate with Cage Fixation for Anterior Cervical Discectomy and Fusion: A Retrospective Analysis of 166 Patients

**DOI:** 10.3390/jcm10143076

**Published:** 2021-07-12

**Authors:** Samuel Sommaruga, Joaquin Camara-Quintana, Kishan Patel, Aria Nouri, Enrico Tessitore, Granit Molliqaj, Shreyas Panchagnula, Michael Robinson, Justin Virojanapa, Xin Sun, Fjodor Melnikov, Luis Kolb, Karl Schaller, Khalid Abbed, Joseph Cheng

**Affiliations:** 1 Department of Neurosurgery, Yale University School of Medicine, New Haven, CT 06519, USA; samuelsommaruga@gmail.com (S.S.); jqcamara@gmail.com (J.C.-Q.); kishan.patel@yale.edu (K.P.); arianouri9@gmail.com (A.N.); shreyas.panchagnula@yale.edu (S.P.); justin.virojanapa@gmail.com (J.V.); xin.sun@yale.edu (X.S.); luis.kolb@yale.edu (L.K.); Khalid.AbbedMD@gmail.com (K.A.); 2 Department of Neurosurgery, Geneva University Hospital, 1205 Geneva, Switzerland; enrico.tessitore@hcuge.ch (E.T.); granitmolliqaj@gmail.com (G.M.); Karl.Schaller@hcuge.ch (K.S.); 3 Department of Neurosurgery, University of Cincinnati College of Medicine, Cincinnati, OH 45267-0515, USA; robinson.michaelw@gmail.com; 4 Yale Center for Green Chemistry and Engineering, New Haven, CT 06511, USA; fjodor.melnikov@yale.edu

**Keywords:** degenerative cervical myelopathy, radiculopathy, ACDF, dysphagia, cervical plating, stand-alone implant

## Abstract

Stand-alone (SA) zero-profile implants are an alternative to cervical plating (CP) in anterior cervical discectomy and fusion (ACDF). In this study, we investigate differences in surgical outcomes between SA and CP in ACDF. We conducted a retrospective analysis of 166 patients with myelopathy and/or radiculopathy who had ACDF with SA or CP from Jan 2013–Dec 2016. We measured surgical outcomes including Bazaz dysphagia score at 3 months, Nurick grade at last follow-up, and length of hospital stay. 166 patients (92F/74M) were reviewed. 92 presented with radiculopathy (55%), 37 with myelopathy (22%), and 37 with myeloradiculopathy (22%). The average operative time with CP was longer than SA (194 ± 69 vs. 126 ± 46 min) (*p <* 0.001), as was the average length of hospital stay (2.1 ± 2 vs. 1.5 ± 1 days) (*p* = 0.006). At 3 months, 82 patients (49.4%) had a follow-up for dysphagia, with 3 patients reporting mild dysphagia and none reporting moderate or severe dysphagia. Nurick grade at last follow-up for the myelopathy and myeloradiculopathy cohorts improved in 63 patients (85%). Prolonged length of stay was associated with reduced odds of having an optimal outcome by 0.50 (CI = 0.35–0.85, *p* = 0.003). Overall, we demonstrate that there is no significant difference in neurological outcome or rates of dysphagia between SA and CP, and that both lead to overall improvement of symptoms based on Nurick grading. However, we also show that the SA group has shorter length of hospital stay and operative time compared to CP.

## 1. Introduction

Degenerative disease of the cervical spine manifests in a wide spectrum of pathologies that encompass disc degeneration, disc herniation, vertebral restructuring, osteophyte formation, and ligamentous hypertrophy [1]. Compression of the neural elements can lead to cervical radiculopathy, myelopathy, or myeloradiculopathy. In the treatment of these pathologies, an anterior, posterior, or combined anterior/posterior surgical approach can be undertaken. However, anterior approaches are often favored for patients with single level disc disease, kyphotic deformity and large focal anterior pathology [2].

**Figure 1:**
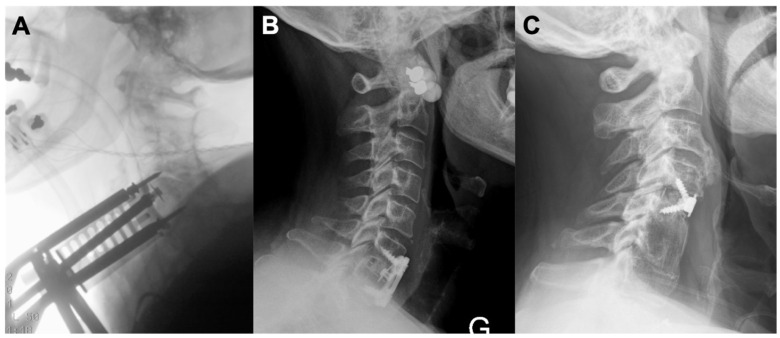
Anterior cervical cage placement and fixation. (**A**) Intraoperative lateral X-ray to assess ACDF cage placement. Casper pins can be seen in the vertebral bodies above and below. (**B**) Post-operative lateral X-ray showing a single level ACDF (HRCC, Eurospine) at C6-C7 secured by a plate (Venture, Medtronic). (**C**) Post-operative lateral X-ray showing a single level ACDF at C4-5 (Zero-P cage, DePuy Synthes) for the treatment of myelopathy due to adjacent segment disease after a fusion with autologous iliac crest bone at C5-6. ACDF = Anterior Cervical Discectomy and Fusion.

Anterior cervical discectomy and fusion (ACDF) is considered the gold standard in the management of cervical disc disease [3,4]. This procedure has been improved on over time since first described by Smith and Robinson in the 1950s, mainly via technological advances. Over the past few decades, ACDF has been coupled with anterior cervical plating (CP), which is thought to reduce the risk of graft extrusion, increase the likelihood of fusion, maintain appropriate lordosis, and reduce the risk of subsidence [5]. In recent years, zero-profile, stand-alone (SA) interbody spacers have been developed as an alternative aimed at decreasing operative time, improving dysphagia complication rates, and preventing adjacent level disease [6], Figure 1. Indications for ACDF with plating or stand-alone cages are variable and are operator dependent, however, the following indications are often used, cases of instability (degenerative or post-traumatic), presence of significant degeneration, concern for fusion failure including in cases of poor bone quality (osteoporosis, rheumatoid arthritis) and tobacco use. In addition, further indication exists with significant alignment correction and increasing number of operated segments [7,8].

There is a dearth of studies in the literature comparing outcomes between SA and CP in single and multilevel ACDF for degenerative cervical disc disease. When compared, the clinical outcomes and dysphagia rates have been either equivocal or not statistically significant [5,9,10,11,12,13]. It remains unclear if this has been due to the limited number patients in these studies and/or the amount of follow-up time.

We sought to study a large North American patient population undergoing ACDF with SA or CP for single and multilevel degenerative disc disease. Herein, we report our experience on a retrospectively collected series of patients with follow-up of up to 24 months after surgery, and describe clinical outcomes and dysphagia rates related to these two techniques. To our knowledge, this cohort represents one of the largest that has been studied in North America.

## 2. Materials and Methods

**Figure 2:**
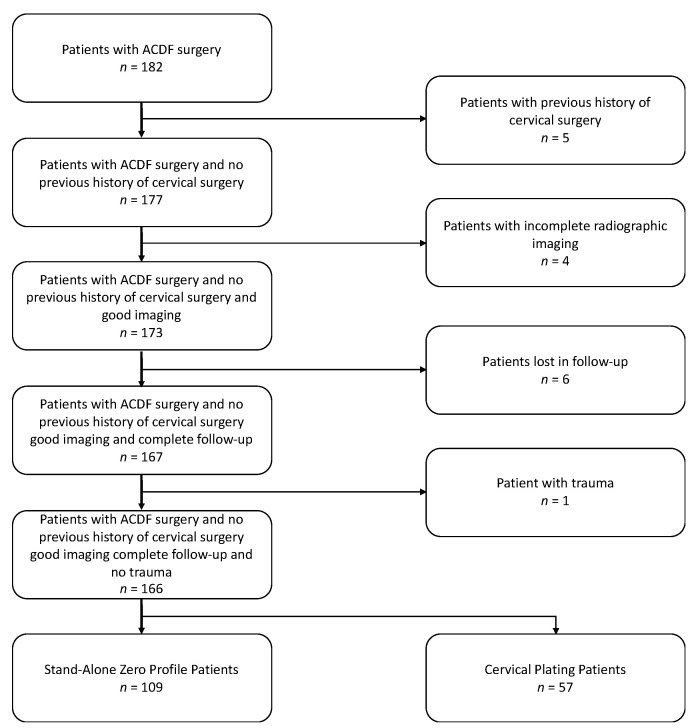
Flow Chart of Patient Cohort. ACDF = Anterior Cervical Discectomy and Fusion.

Between January 2013 to December 2016, a total of 182 patients underwent single to three-level ACDF in the Department of Neurosurgery at Yale New Haven Hospital, New Haven, CT, USA. Of the 182 patients, we retrospectively reviewed 166 consecutive patients that had received ACDF (Figure 2). Yale University’s IRB approved the protocol for this study (protocol number 200020713), and the study was exempt from informed consent. Inclusion criteria included: signs and symptoms of cervical radiculopathy, myelopathy, or myeloradiculopathy, unresponsiveness to conservative measures, ages between 18–85, and disc herniation identified by MRI with evidence of nerve root and/or cord compression. Exclusion criteria included: patients presenting with ossification of the posterior longitudinal ligament, a history of malignancy, evidence of systemic or local infection, history of cervical spine trauma, prior cervical spine surgery, and patients requiring simultaneous anterior and posterior surgery. Demographic information, medical comorbidities, selected medications and selected personal history were also collected.

### 2.1. Surgical Procedure

All surgical procedures were performed by two senior spine surgeons using the standard Smith-Robinson approach on the patient’s right side. After sterile prep and draping, a right-sided transverse incision was made with a #10 blade. The platysma was transected using bovie electrocautery. Fascial planes under the platysma were exposed cranially and caudally using Metzenbaum scissors. A corridor was then made in between the sternocleidomastoid muscle and strap muscles, preserving their respective fascial planes. Using Cloward hand-held retractors, the esophagus was protected medially and the carotid laterally. The prevertebral fascia was dissected off the ventral spine, exposing our index disc level(s) and longus colli bilaterally. Once our index disc level(s) were confirmed with a needle and fluoroscopy, bilateral colli were dissected of the lateral ventral spine using bovie electrocautery. Appropriately sized retraction blades with teeth were placed under the dissected longus colli to retract over the longus colli away from our working field. Caspar pins were then applied to the vertebral bodies above and below to help distract our disc space. Disc was removed with great care taken in removing the cartilaginous endplate to avoid disruption and damage of the bony integrity of the endplates. Posterior osteophytes were drilled using a high-speed cutting burr in combination with Kerrison rongeurs. After decompression of the spinal cord and nerve roots, the appropriate size cage was selected using trial spacers under fluoroscopic guidance to ensure good height and width as well as to not over-distract the facet joints. We used demineralized bone matrix to pack all interbody spacers. For patients undergoing SA device insertion (Globus Coalition AGX, Audubon, PA, USA), the implant was placed into the intervertebral disc space and a pilot hole was drilled at the rostral and caudal endplates under fluoroscopic guidance and this was followed by screw insertion. For patients undergoing CP devices, the spacer was inserted under fluoroscopic guidance followed by positioning of a 4-hole plate across the midline of rostral and caudal ventral vertebral bodies. Pilot holes were drilled into rostral and caudal vertebral bodies followed by screw insertion, again using fluoroscopy to ensure proper placement.

### 2.2. Clinical Measures

The operative procedure details such as the post-operative symptoms, the post-operative Nurick grade, the number of index levels, operating time, blood loss, presence of a CSF leak, and hospital length of stay were all collected. Nurick grade scores were collected on pre-op, 2 weeks, 3-, 6-, 12-months, and up to 24-months or last follow-up. Neurological outcome was dichotomized in the Nurick grade. Outcome measures included Bazaz dysphagia score at 3 months [14]. We defined optimal outcome as an improvement in the Nurick grade. We defined suboptimal outcome as no change or a decline in Nurick grade.

### 2.3. Statistical Analysis

Statistical analysis was performed with R-studio Desktop v1.1.383 (R-studio, Boston, MA, USA) programming software. Continuous variables are presented as mean (standard deviation (SD)) or median (interquartile range (IQR)), while discrete variables are presented as count (percentage (%)).

Assessments of potentially significant differences between patients with optimal and suboptimal outcomes groups were performed using the Fisher exact test for categorical variables and the Mann-Whitney U test for continuous variables. 

We constructed a multivariable logistic regression model by including variables that reached a predetermined significance level of *p* < 0.2 in univariate analysis. Additionally, universal confounders and other variables selected based on expert opinion were forced into the model, including Nurick grade and number of levels of surgery. Covariates with *p* > 0.1 were removed and collinearity was assessed based on variance inflation factor. A 2-sided *p* value of <0.05 was used to determine which variables were independently associated with an optimal outcome.

## 3. Results

### 3.1. Demographics 

Our baseline patient population characteristics are reported in Table 1. A total of 166 patients (92 females and 74 males) underwent ACDF and met our inclusion criteria, with age ranging from 23 to 85 (mean 53 years). Of the 166 patients, 92 (55%) presented with radiculopathy, 37 (22%) with myelopathy, and 37 (22%) with myeloradiculopathy. In comparing SA and CP patients, 61% and 46% suffered from radiculopathy, 14% and 39% from myelopathy, and 26% and 16% from myeloradiculopathy, respectively. 

Twenty-four (14%) of our patients had diabetes, 45 (27%) were current tobacco users, 47 (28%) had a history of tobacco use longer than 1-year and 3 patients (2%) were formally diagnosed with osteoporosis. For preoperative pain management, 61 (37%) patients were currently using opioid medications, 40 (24%) were on Gabapentin or Pregabalin, 58 (35%) were using NSAIDs for over 3 months, 54 (33%) were on antidepressants, and 13 (8%) were taking steroids chronically for medical conditions. In addition, 98 (59%) patients were actively involved in physical therapy and 22 (13%) had at least one epidural steroid injection.

Among the 166 patients, 109 underwent ACDF with SA devices (66%) and 57 underwent ACDF with CP (34%). Eighty-five patients (51%) underwent 1 level ACDF; 65 patients (39%) underwent 2 levels and 16 patients (10%) had 3 levels. There was no difference in the number of levels of surgery between the SA and CP groups (Table 2). The average duration of surgery for all ACDF procedures was 150 (±64) min, with 194 (±69) and 126 (±46) min for CP and for SA respectively (*p* < 0.001). The blood loss was minimal; only 4 patients (2%) had more than 100 mL of blood loss. Only one patient had a CSF leak. The average length of hospital stay for the total population was 1.7 ± 1 days, with the SA group having a shorter length of stay compared to the CP group (1.5 ± 1 vs. 2.1 ± 2 days) (*p* = 0.006).

### 3.2. Clinical Outcomes

The mean follow-up for the SA and plate groups was 7.5 months and 10.6 months, respectively, with an overall mean follow-up of 8.6 months. Of the 166 patients, 82 (49%) had a post-surgical follow-up appointment at 3 months to assess for dysphagia. Three patients had mild dysphagia and none of them had moderate or severe dysphagia. However, all of these patients recovered with no further consequences at the last follow-up. As shown in Table 3, the mean Nurick score for the myelopathy and myeloradiculopathy groups improved regardless of the surgical technique or number of index levels. The baseline Nurick score was a score of 1 for 21 patients (28%), a score of 2 for 41 patients (55%), a score of 3 for 8 patients (11%), a score of 4 for 3 patients (4%) and a score of 6 for 1 patient (1%). The Nurick score at last follow up was 0 for 55 patients (74%), 1 for 12 patients (16%), 2 for 5 patients (7%), 3 for 1 patient (1%), and 6 for 1 patient (1%). Overall, 63 patients improved their Nurick score (defined as an optimal outcome) while 11 patients showed no change or decline in their Nurick score (defined as a suboptimal outcome). 

Multivariable analysis (Table 4) revealed that length of stay is a statistically significant independent predictor of suboptimal outcome. Prolonged length of stay was associated with reduced odds of having an optimal outcome by 0.50 (95% CI = 0.35–0.85, *p* = 0.003). Moreover, the SA technique was not found to be an independent predictor of better outcomes.

## 4. Discussion

ACDF is a well-established surgical treatment for anterior degenerative cervical pathology. ACDF is often done with the use of an anterior vertebral body plate, with the goal of maintaining stability, promoting fusion, preventing graft extrusion, preventing graft subsidence, and maintaining desired cervical lordosis. Potential instability in both degenerative and traumatic cases is a common reason for the addition of additional structural support via plate fixation. However, a known morbidity of ACDF with cervical plating is post-operative dysphagia, ranging from 2 to 67% in the post-operative period [15]. With the goal of reducing dysphagia and other perioperative morbidities, stand-alone (SA) ACDF systems were developed. Additional potential benefits of SA devices include that they can provide lordotic correction and are anchored with screw fixation. The latter aspect may be relevant in patients with segmental degenerative instability. 

Despite the introduction of stand-alone cages as an alternative to cervical plating, clinical outcomes appear to be similar between the two groups. A systematic review by Cheung et al. of 19 studies comparing ACDF with a cage-only technique and conventional cage-plate technique found that stand-alone cage was associated with less dysphagia, intraoperative blood loss, and adjacent segment disease. However, the stand-alone cage was also shown to have increased rates of subsidence and less restoration of cervical lordosis [5]. Similarly, a recent meta-analysis by Gabr et al. demonstrated that stand-alone anchored spacers were associated with less dysphagia compared to the plate-screw construct [16]. Lastly, a systematic review by Boer et al. showed that there was no difference in clinical (visual analog scale, neck disability index) or radiological (cervical lordosis and fusion) outcomes between the two groups, but stand-alone devices were associated with shorter operative time [17].

The pathogenesis of dysphagia in ACDF has not been clearly elucidated. Previous studies have implicated that injury to the esophagus, soft tissue edema, localized bleeding, and adhesions surrounding the CP may contribute to post-operative dysphagia [15]. Indeed, removal of the anterior plate and lysis of associated adhesions has been shown to clinically improve patients’ experience of dysphagia both immediately after surgery and at later timepoints [18]. A study by Lee et al. also noted a correlation between the thickness of the cervical plate and post-operative dysphagia, suggesting that physical obstruction may also play a role [19]. 

In addition to demonstrating no statistically significant difference in morbidity of SA and CP with respect to post-operative dysphagia, our study also found no significant difference in intraoperative blood loss. This data differs slightly from the results described in Cheung et al., which suggest that a cage-only technique is associated with less intraoperative bleeding. However, it is important to note that the average blood loss of ACDF is quite low, so the difference that was noted between SA and CP is not likely to be clinically significant [5]. We also found a significant reduction in operative time with cage-only implants, as well as a decrease in hospital length of stay. Consistent with other studies comparing SA to CP, our data support that either intervention confers optimal outcomes in patients with degenerative cervical myelopathy or radiculopathy. Based on Nurick scores postoperatively, 85% of patients treated in our study had improvement in neurological symptoms, social independence, walking ability, and ability to work full time, with no significant difference in outcomes between SA and CP.

Given our findings with respect to morbidity, there is no clear advantage of stand-alone cage over cervical plating. The stand-alone cage was not found to be a predictor of superior outcomes. Based on these results, it will be important in future studies to investigate the cost-effectiveness of CP vs. SA in anterior cervical discectomy and fusion; in the setting of similar surgical outcomes, as was reported in our study, the more affordable option should be pursued.

An important point to note is that while anterior approaches are indicated in patients with kyphosis, it is unclear how the choice of these 2 techniques affects surgical decision-making. Some may prefer stand-alone cages in such instances given that they can provide different degrees of lordosis. On the other hand, if the implicated disc is present adjacent to a kyphotic segment, increasing lordosis at that level may increase segmental kyphosis at the adjacent level. Further research is warranted with regards to whether these 2 techniques carry different risks and benefits with regards to patients with cervical kyphosis or malalignment.

## 5. Limitations

There are important limitations with our study. First, patients were not randomized to treatment modality. The two surgeons in this study had different preferences in using SA vs. CP, such that one surgeon used only SA while the other used only CP in their practice. Thus, outcomes and operative time may be biased by surgeon experience, number of years in practice, and technique. However, this also eliminates selection bias, as patients were not selected to receive SA versus CP based on any preoperative parameters or surgeon preference, as each surgeon exclusively performs ACDF with SA or CP. Second, this study does not include a power analysis to determine if the sample size in adequate. Nonetheless, it is important to note that this cohort represents one of the largest studies that examines surgical outcomes between SA and CP, and is one of few in North America. Third, our study warrants a cost analysis between the two approaches to further identify which is more favorable if no change in morbidity is observed. Moreover, if dysphagia avoidance is not an indication for SA grafts, other variables such as the relationship of operative time and blood loss, or graft extrusion, pseudoarthrosis, subsidence, and sagittal alignment should be further assessed between SA and CP techniques to further understand the advantages of the SA technique. Fourth, the Bazaz criteria were used in this study rather than other metrics, such as the EAT assessment, which have been validated as an outcome tool for dysphagia. Lastly, because this study is a retrospective analysis, we were unable to assess the presence of dysphagia in every patient included in this study, due to inconsistent reporting in the absence of symptoms.

## 6. Conclusions

Patients undergoing ACDF with SA had significantly decreased hospital length of stay and operative time compared to patients with CP. The type of procedure did not affect neurological outcome based on Nurick grade. Hospital length of stay was found to be a predictor of a poor outcome regardless of technique. In general, both ACDF with CP and SA are effective treatment options and provide comparable outcomes. 

## Figures and Tables

**Table 1:** Population Characteristics.

Covariate	All Patients *n* = 166	Stand Alone *n* = 109	Cervical Plate *n* = 57	*p*-Value
Demographics				
Age, *n* (SD)	53 (13)	52 (14)	54 (13)	0.50
Female, *n* (%)	92 (55)	66 (61)	26 (46)	0.07
Medical Comorbidities				
BMI, mean (SD)	29 (9)	28 (6)	30 (6)	0.05
Diabetes Mellitus, *n* (%)	24 (14)	15 (14)	9 (16)	0.73
Current Smoker, *n* (%)	45 (27)	30 (28)	15 (26)	0.87
Former Smoker, *n* (%)	47 (28)	24 (22)	23 (40)	0.03
Osteoporosis, *n* (%)	3 (2)	3 (3)	0	0.08
Treatments, *n* (%)				
Number of Medication, mean (SD)	6 (4)	6 (4)	6 (4)	0.72
1 opioid	50 (30)	32 (29)	18 (32)	0.77
2+ opioids	11 (7)	8 (7)	3 (5)	0.60
1 depression medication	44 (27)	33 (30)	11 (19)	0.11
2+ depression medications	10 (6)	7 (6)	3 (5)	0.76
Pregabalin or Gabapentin	40 (24)	30 (28)	10 (18)	0.17
Chronic NSAID	58 (35)	40 (37)	18 (32)	0.44
Chronic Steroid	13 (8)	10 (9)	3 (5)	0.60
Physical Therapy	98 (59)	67 (61)	31 (54)	0.52
Epidural Steroid Injection	22 (13)	17 (16)	5 (9)	0.25
Clinical presentation, *n* (%)				
Radiculopathy	92 (55)	66 (61)	26 (46)	0.07
Myelopathy	37 (22)	15 (14)	22 (39)	0.001
Myeloradiculopathy	37 (22)	28 (26)	9 (16)	0.13

NSAID = Non-steroidal anti-inflammatory drugs, SD = Standard deviation, BMI = Body mass index.

**Table 2:** Surgical Details.

Covariate	All Patients	Stand Alone	Cervical Plate	*p*-Value
Number of Levels of Surgery				
1	85 (51)	59 (54)	26 (46)	0.30
2	65 (39)	37 (34)	28 (49)	0.63
3	16 (10)	13 (12)	3 (5)	0.13
Levels of Surgery, *n*				
C2-C3	25	13	12	0.15
C3-C4	66	42	24	0.66
C4-C5	133	88	45	0.79
C5-C6	133	89	44	0.51
C6-C7	68	48	20	0.26
C7-T1	52	35	17	0.76
Total number of levels	477	315	162	
Length of Surgery, mean (SD)	150 min (64)	126 min (46)	194 min (69)	<0.001
Blood Loss, *n* (%)				
0–50 mL	132 (80)	85 (78)	47 (82)	0.49
51–100 mL	30 (18)	24 (22)	6 (10)	0.05
>100 mL	4 (2)	0	4 (7)	0.05
CSF leak, *n* (%)	1 (1)	0	1 (2)	0.32
Length of stay, mean (SD)	1.7 (1)	1.5 (1)	2.1 (2)	0.006
Dysphagia at 3 months, *n* (%)				
None	79 (96)	52 (98)	27 (93)	0.34
Mild	3 (4)	1 (2)	2 (7)	0.34
Moderate	0	0	0	N/A
Steroid use, *n* (%)	10 (6)	9 (8)	1 (2)	0.07

**Table 3:** Surgery Outcomes for Myelopathy and Myeloradiculopathy Cohorts.

Patient Group	All Patients (*n* = 74)		Stand Alone (*n* = 43)		Cervical Plate (*n* = 31)	
Nurick Score	Baseline	Last Follow-Up	Baseline	Last Follow-Up	Baseline	Last Follow-Up
0	0	55 (74)	0	36 (84)	0	19 (61)
1	21 (28)	12 (16)	11 (26)	3 (7)	10 (32)	9 (29)
2	41 (55)	5 (7)	25 (58)	4 (9)	16 (52)	1 (3)
3	8 (11)	1 (1)	6 (14)	0	2 (7)	1 (3)
4	3 (4)	0	1 (2)	0	2 (7)	0
5	0	0	0	0	0	0
6	1 (1)	1 (1)	0	0	1 (3)	1 (3)

**Table 4:** Multivariable Analysis.

Covariates	OR (95% CI)	*p*-Value
Patient Characteristics		
Age	0.99 (0.97–1.04)	0.79
Sex (Female)	1.21 (0.27–2.30)	0.71
Diabetes	0.44 (0.12–1.23)	0.14
Surgery Characteristics		
Stand-Alone Zero Profile	0.67 (0.14–1.61)	0.49
Length of Stay	0.5 (0.35–0.85)	0.003
Number of levels of Surgery		
1 level	1	NA
2 levels	1.99 (0.15–1.70)	0.22
3 levels	3.56 (0.77–21)	0.25

OR = Odds Ratio, CI = Confidence Interval.

## Data Availability

Data available on request (not publicly available for privacy protection).
